# Tennessee State Medical Society

**Published:** 1891-06

**Authors:** Frank Trester Smith


### TENNESSEE STATE MEDICAL SOCIETY.

The fifty-eighth annual meeting of this Society was held in Nashville, April 14th, 15th and 16th. The meeting was one of the largest in the history of the Society, there being about 150 delegates in attendance.

The following exhibits were made : Horlock's Malted Milk, Tarrant & Co.'s Hoff Malt Extract, Fairchild Bros. & Foster's Digestive Ferments.

On the first day, after the usual preliminaries and invitations to visit various institutions, the Secretary presented his report which showed a membership of 342.

The Treasurer's report showed that the Society was in a good financial condition. There was some difference between the Treasurer and the Secretary, owing to the fact that the latter had received dues from certain members who should have been suspended in accordance with the by-laws. This created quite a breeze, but the matter was finally adjusted amicably.

Dr. Frank Trester Smith appealed from the Publishing Committee on account of their leaving out of the transactions of

1889 the description of certain inventions which the Society at that meeting ordered to be printed, but which, through some misunderstanding, were omitted.

The paper was referred to the new Publication Committee.

Dr. J. F. Grant was elected an honorary member for life. Dr. Grant was a former president of the Society.

Dr. Thos. L. Lipscomb, of Shelbyville, was invited to a seat on the right of the President. He is one of the oldest members of the Society, having missed but few of its meetings since the organization fifty-eight years ago.

One of the most enjoyable sessions was that of the night of the first day, when a musical and literary feast was spread for the benefit of the Society. Dr. J. D. Plunkett, of the Committee of Arrangements, presided, and announced that the meeting was held for the purpose of welcoming the doctors to the city. After prayer by the Rev. Jerry Witherspoon and some instrumental music, Mayor Lutterer was introduced, who welcomed all to Nashville.

Mrs. A. H. Stewart then sang "Heaven Hath Shed a Tear," which was well received and encored.

Gen. H. H. Norman then welcomed the Society on behalf of the Governor who was prevented from being present on account of illness. Judge J. M. Dickson was then introduced, and welcomed the Society on behalf of the citizens. Then followed the address of the evening by the President of the Society, Dr. Geo. A. Baxter, of Chattanooga : "Topics of Import to the Medical Profession." The above interspersed with music, instrumental and vocal, formed a most enjoyable programme.

The following is a list of papers read at the various sessions :

Report of State Board of Medical Examiners.—T. J. Happel, Trenton.

Ovulation and Menstruation.—Geo. R. West, Chattanooga.

Preparatory Treatment of Parturient Women.—C. W. Beaumont, Clarksville.

Posology.—W. M. Vertrees, Nashville.

Treatment of Wounds of the Cranial Sinuses.—W. T. Briggs, Nashville.

A Plea for Early Operative Interference in Ovarian Tumors. —J. H. Blanks, Nashville.

The Treatment of Pneumonia; Past and Present Methods; Has the Mortality been changed?—Thos. M. Woodson, Gallatin.

Phthisis Pulmonalis; with Special Reference to Prophylaxis.—J. R. Buist, Nashville.

The Anatomy and Pathology of the Ileo-Cæcal Region.—Richard Douglass, Nashville.

Urethral Stricture.—J. W. Handley, Nashville.

Abscesses.—T. J. Happel, Trenton.

Has Progress been made in the Treatment of Typhoid Fever? —C. M. Sebastian, Martin.

Chronic Endometritis.—J. S. Cain, Nashville.

Retention of the Placenta of Miscarriage; How Shall We Treat such Cases.—A. J. Swaney, Nashville.

Indigo as an Emmenagogue.—J. L. Jones, Bells.

Diabetes.—J. A. Witherspoon, Columbia.

Dr. W. Whitford, Chicago, acted as official stenographer.

The following officers were elected:

President—J. W. Penn, Humboldt; Vice-Presidents—J. A. Witherspoon, Columbia; C. H. Lovelace, Dukedom, and C. E. Ristine, Knoxville; Secretary—D. E. Nelson, Chattanooga; Treasurer—J. P. C. Walker, Dyersburg.

The next meeting will be held in Knoxville, on the second Tuesday in April, 1892. FRANK TRESTER SMITH.

---

NATIONAL STERILITY.—In France in 1888 there were 882,-639 births and 794,933 deaths. The rate of births has fallen from 30 per 1,000 in the early years of the century to 23 per 1,000. The number of marriages has fallen to 7.1 per 1,000, and the number of births to each family has fallen to 3. Divorces are increasing in frequency, especially among the educated classes, while the tendency is for marriage to take place later in life.—*The Journal, Little Rock.*

